# Development of fully coupled deviated well drill string dynamics simulation model for fatigue and vibration analysis

**DOI:** 10.1177/00375497261465960

**Published:** 2026-07-13

**Authors:** Sampath Liyanarachchi, Geoff Rideout

**Affiliations:** 1 Department of Mechanical Engineering, Memorial University of Newfoundland, St. John’s, Canada

**Keywords:** Drill string vibrations, fatigue prognosis, directional drilling, bond graph method, multibody dynamics

## Abstract

Drilling is an expensive but necessary part of mining, geothermal and oil operations. The cost of drilling can be reduced using simulation models to optimize parameters and perform fatigue prognoses, expanding the useful life of downhole equipment. However, current state-of-the-art simulation models have limitations in modeling drill string dynamics in the build section and generating stress history for drilling and tripping operations. This paper presents a model with an efficient contact algorithm, reconfigurable stiction/friction submodels, accurate boundary conditions, expanding drill string length as the bit advances and readily available stress history for any component. The model predicts the onset and location of wellbore contact, as well as axial, lateral and torsional vibrations in any well section. Multiple well trajectory simulations are presented, showing responses such as whirling and snaking. Stress histories and predicted fatigue life of components are compared, showing how the model can be used for selecting the best well trajectory and drilling parameters during well planning. These case studies also show that dogleg severity and kick-off point of the deviated section influence the fatigue life of the downhole components.

## 1. Introduction

Improving efficiency and reliability in drilling, especially for extended-reach wells, will be beneficial in meeting future global energy demands by enabling mineral, petrochemical and geothermal energy extraction.

The oil and gas industry is essential for the global economy, and it impacts everything from heating to electricity, transportation and manufacturing.^1^ Despite the environmental impact and government policies, the global oil demand is expected to rise by 6% between 2022 and 2028 to reach 105.7 million barrels per day.^2^ To meet these demands and to identify potential future reservoirs, the drilling industry is projected to grow from USD 9.82 billion in 2020 to USD 17.44 billion in 2027.^3^ Technologies such as directional drilling have facilitated attaining these goals by enabling access to hard-to-reach reservoirs and improving extraction efficiency. Moreover, directional drilling enables accessing multiple oil deposits from a single well, reducing surface disturbance and environmental impact.

Another drilling application is geothermal, an inexhaustible, renewable energy source with minimal environmental impact and a smaller surface footprint. Recent developments in deep geothermal and closed-loop geothermal using U-shaped directional wells have shown the potential to be accessible anywhere in the world.^4^ Yet, geothermal power generation is still not widely adopted due to the high initial cost. It is estimated that around 50% to 75% of geothermal development cost is tied up in drilling and well construction.^5^

Simulation models help the drilling process by optimizing the well parameters and identifying harmful vibration sources. Moreover, having accurate simulation models will facilitate failure prediction of downhole equipment, reducing fishing and unplanned tripping operations. Wash-out and twist-off are the two general forms of mechanical failure in drill pipes, and they are caused by fatigue loading.^6^ The main reasons for fatigue failure are complex loading, combined stresses and different types of vibrations. Hence, simulation models are needed to calculate internal stresses for fatigue calculations. However, current models have limitations, especially when simulating deviated wells.^7,8^

Due to inaccuracies in fatigue prognosis, the useful life of a drill pipe is usually calculated based on the number of times or hours it has been used. These calculations are often conservative, and drill pipes are replaced with significant usable hours remaining to avoid unexpected pipe failures.^9^ This can be a significant cost, especially considering that the drill pipe market is forecast to reach US$1.6 billion by 2027.^10^ Even though a fast, reliable and accurate directional drilling vibration simulation model is motivated, most current dynamic simulation models in the literature are either limited to vertical or horizontal sections of the wellbore or rely on limiting assumptions and simplifications.

This paper aims to improve the state of the art by incorporating an efficient contact model into a fully dynamic three-dimensional (3D) vibration model with realistic boundary conditions, stick/slip friction, the ability to increase string length as penetration progresses and stress history generation for fatigue life prediction of components.

Section 2 of this paper provides background information about drill string failure modes and simulation models. Section 3 provides a detailed description of the drill string dynamic model; this includes developing a friction model, implementing the contact algorithm, and a method to continually increase string length and guide the bit as penetration progresses. Section 4 discusses three case studies and the potential utilization of the model. Whirling vibrations in horizontal well sections were analyzed to verify the model’s behavior and investigate and compare the computational speed of friction models. In the second case study, three different well profiles were analyzed to show the procedure of extracting internal stresses and performing fatigue analysis. This shows how the model can help compare candidate well trajectories. The third case study provides a durability analysis of a downhole drill pipe in the horizontal section and compares how relative distance from the bit affects its lifespan. The conclusions and future work are given in Section 5.

## 2. Literature review

Three main topics are covered in this section. First, a detailed summary of drill string failure modes is provided. Next, existing vibration models are reviewed with a focus on their ability to predict dynamic responses and stress histories in deviated wells. Because most drill string failures occur due to fatigue, the third subsection of the literature review addresses fatigue damage prediction methods.

### 2.1. Drill string failure modes

The drill string is a long, slender structure that transfers torque from the top drive or kelly bushing into the bit and provides a path for transferring the cutting fluid through the well. Most of the drill string’s length consists of thin-walled drill pipes with low bending and torsional rigidity. The remaining portion is called the bottom-hole assembly (BHA), typically a few hundred meters of heavy-duty drill pipes, drill collars, stabilizers, directional drilling instruments, mud motors and the bit. Unlike the drill pipe section, which is in tension during drilling operations, this section is comparatively rigid and subjected to compressive load.

The drill string operates in a harsh and unpredictable environment and is subjected to complex loads and stresses. Due to these conditions, there are many challenges, such as excessive pipe wear, sticking, twist-off, burst-off, wash-off and failure in downhole measuring tools. Even though the causes and failure methods are known, and significant progress in manufacturing and analysis techniques has been achieved in recent years, the occurrence of drill string failure is still significant.^11^ Most of the excessive loads and stresses applied to the drill string are caused by uncontrolled vibrations, which lead to fatigue failure in drill pipes, unscrewing of threaded connections, premature failure of downhole equipment, reduction in drilling performance and drill bit tooth fracture.^12^

Vibrations during drilling are unavoidable since crushing or chipping rock is a destructive process that uses a substantial amount of energy.^13,14^ In addition, imbalances and bends in the rotating drill string and operating conditions of drill string equipment, such as the top drive, can generate vibrations, which can be difficult to control due to the low stiffness of the drill string. Uncontrolled string vibration increases the tendency for downhole equipment failure, which drives up drilling costs by 2% to 10% beyond initial estimates and necessitates expensive and time-consuming tripping/fishing operations.^15,16^ Also, drill string vibrations can reduce drilling efficiency and fatigue life. Therefore, understanding the sources and type of drill string vibrations will be beneficial for predicting fatigue life, optimizing drilling operations and developing novel solutions for vibration reduction.

Drill string vibration can be divided into three main types: axial, torsional and lateral.^16^ Axial vibration in its most severe form is called bit bounce, which can momentarily force the bit to lose contact with the formation. However, in most cases, there is not enough energy to lift the drill string off the bottom and bounce; instead, shocks created due to the cutting action will be transmitted up the drill string. These shocks can be detected as harmonic variations in weight-on-bit (WOB) and will cause broken teeth and reduce rate of penetration (ROP).^17^ Roller cone bits are generally known for axial vibrations at the drilling speed, and tri-cone bits tend to excite axial vibration at three times the rotational frequency.^18,19^ Shock subs are placed above the bit to reduce axial vibrations, acting as vibration absorbers. Another way to reduce the severity of axial vibrations is by increasing the WOB. However, this might cause other issues, such as stick-slip.^13^

Bit-rock forces easily excite vibration in the highly compliant torsional modes, causing increased bit wear and fatigue damage to BHA components. The most severe form of torsional vibration is stick-slip, which happens when the bit digs into the formation deeply enough to slow it down or completely stop the bit.^20^ This causes a winding effect in the drill string followed by release and overspeed, leading to fatigue damage and loosening of threaded connections.^21^ Stick-slip conditions are generally observed when drilling hard formations using Polycrystalline Diamond Compact (PDC) bits.^22^ To mitigate stick-slip, drillers will reduce the WOB and increase the rotating speed.^23^ However, this will have a negative impact on the drilling performance and can cause lateral/axial vibration problems.^24^ Therefore, active control systems have been developed to reduce torsional vibrations, and accurate vibration simulation models will be beneficial when developing such algorithms.^25–27^

Whirling is another vibration mode found in drill strings, and it is considered the main reason for drill string fatigue failure.^28^ Whirling will cause lateral motion in the drill string, resulting in high-frequency bending moment fluctuation and damage in BHA components.^29^ Bit whirl happens when the bit cuts into a hole larger than its diameter, allowing it to wander around the wellbore instead of rotating around the center of the well. Moreover, this over-gauged hole will further increase side cutting and the tendency to whirl.^30^ BHA whirl is another type of drill string whirl, and it is caused by the eccentric rotation of the drill string around a point other than its center.^31^ Based on the center of the motion of the whirl and the rotational direction of the drill string, whirling can be categorized into three main types: forward, backward and chaotic. In forward whirl, the center of the motion rotates in the same direction as the drill string, and in backward whirl, the opposite is true.^32^ Chaotic whirl is a combination of the forward and backward whirl and is considered the most destructive.^33^

### 2.2. Existing vibration simulation models

Knowing what is happening down the hole is important to prevent damage to components and improve drilling performance. Vibration measurement tools have significantly improved in recent years, moving away from early mud-pulse telemetry methods. Li et al.^33^ and Cai et al.^34^ provide a detailed review of current trends in high-fidelity downhole vibration measurements. Despite these improvements, several challenges, such as transmission constraints and high equipment cost, have hindered industry-wide adoption of vibration measurement tools. Instead of deploying downhole vibration-measuring equipment, simulation models can serve as “soft sensors” using surface-measured parameters.^35^

Developing accurate simulation models has been difficult, especially for deviated wells, due to several challenges, including complex intermittent contact between the drill string and the wellbore, nonlinearities in bit-rock interactions, high computational requirements and limited availability of downhole measurements for validation.

Millheim and Apostal^36^ developed a dynamic model of the BHA using 3D finite element analysis (FEA). The model included the inertia forces from the drill string, buoyancy force from drilling mud, and bit side force from bent subs and whipstocks. Due to computational limitations, this model was limited to the BHA and did not include vibrational analysis of the drill string. Mitchell and Allen^37^ used FEA to identify critical rotational speeds of eight case studies where BHA failure had occurred and matched the operating condition at the time of failure, showing the importance and capabilities of vibration simulation models.

Christoforou and Yigit^38^ developed a fully coupled torsional-axial-bending vibration model to be used in developing an active control strategy for stick-slip vibrations for vertical wells, and this model was further improved by Yigit and Christoforou^24^ by including the dynamics of the rotary table and the drive motor. Afterward, by including a nonlinear friction-based cutting model, Kamel and Yigit^17^ further expanded the previous model. Chen et al.^39^ developed an intelligent stick-slip control strategy using real-time BHA and vertical drill string vibration data generated from a simulation. Kreuzer and Steidl^40^ stated that torsional vibrations reduce the ROP and may lead to failure in drilling equipment. This research then developed a control strategy for vertical drill strings based on decomposing string dynamics into two traveling waves, starting from the top drive and the bit. de Moraes and Savi^41^ developed a four-degrees-of-freedom vertical drill string vibration simulation model that considered axial-torsional-lateral coupling, nonsmooth contact, a bit-rock interaction model with a time-delayed approach, eccentricity and hydrodynamic forces. All the research work mentioned in this paragraph depicts the benefit of simulation-based design when developing an active control strategy for stick-slip vibrations for vertical wells.

Ghasemloonia et al.^42^ developed a coupled axial-transverse vibration simulation model using explicit FEA to model the BHA and Euler-Bernoulli beam theory to model a vertical drill string. The aim was to analyze the effect and fatigue damage of passive vibration-assisted rotary drilling (pVARD) tools. Rideout et al.^43^ have developed a simulation model for a vertical well utilizing the bond graph (BG) approach, where the drill string is divided into rigid segments and has connected springs between them to represent the drill string’s torsional, axial and bending stiffness. Furthermore, Sarker et al.^44^ improved this model to analyze bit bounce, stick-slip and whirling conditions. However, Sarker’s simulation model uses a soft string contact model based on the research work done by Aadnoy and Djurhuus^45^ and does not include lateral vibration in the build section due to the challenges in modeling clearance between pipe and transitioning wall section. Similarly, Xie et al.^46^ developed a dynamic drill string model for the horizontal well section for analyzing longitudinal, lateral and torsional vibrations, and this model included bit-rock interactions and nonlinearities for intermittent contact between the drill string and well bore. However, it did not include the dynamics of the build section due to mathematical constraints of the modeling technique, including difficulty in calculating the variation force and equlibrimum position of rigid segments within the build section. Ritto et al.^47^ analyzed dynamics of horizontal drill strings using the bar model discretized by means of the FEA method and proposed a stochastic model for the friction coefficient with an autocorrelation function. Volpi et al.^48^ further investigated coupled lateral-torsional vibrations to identify possible safe operation regions. Moharrami et al.^49^ developed a stick-slip analysis model using a finite element approach and showed that the self-excited torsional vibration could occur below the first fundamental natural frequency of the vertical drill string, depending on the operational parameters.

All the simulation models discussed up to this point were limited to vertical or horizontal sections of the well, and none of those models considered the dynamics in the build section or complete treatment of the drill string with a compatible transition between vertical, build and horizontal sections due to modeling limitations and difficulties in calculating contact points and normal forces in the build section. Liu and Gao^50^ developed a nonlinear dynamic model for deviated wells with contact and fluid damping effects using Lagrangian dynamics and solved using a Runge–Kutta solver. That research work also speculated that increasing the inclination will cause an increase in drill string wear and the likelihood of key seating. Aarsnes and Shor^51^ studied the torsional vibrations under off-bottom conditions in deviated wells to model the stick-slip phenomena observed in field data after connections and back-reaming operations. Modeling dynamics in deviated wells is difficult, and to reduce this complexity, many models, including the two research works mentioned in this paragraph, have limited the profile of the well to a single plane.

Yang et al.^52^ developed a multidirectional coupled drill string vibrational model to predict the deviation in well trajectories. This model does not focus on modeling drill string vibrations but has included them in the well trajectory calculations to improve accuracy. Tengesdal et al.^53^ developed a real-time three-dimensional drill string dynamic model based on a parametric curve and lumped-parameter modeling where Kane’s method is used to establish the equations of motion. In this model, the drill string is assumed to be in the center of the wellbore curve and always in contact with the bottom wall of the wellbore, which might result in inaccuracies for highly deviated well profiles.

Another approach to handle the modeling complexity of deviated wells is using FEA-based dynamic drill string models. Li et al.^54^ have commented on the current inadequate understanding of the lateral vibration models for air drilling in highly deviated wells and have developed a dynamic model using FEA methods and beam-column theory. Cai et al.^55^ developed a nonlinear lateral vibration model using the beam FEA method for the curved well’s vertical, horizontal and build sections and validated it using field data. As a case study, that research proposed an optimal length for a given well design and discussed the influence of parameters such as WOB, friction and stabilizers on drilling performance. Tran et al.^56^ developed a 3D beam FEA model to analyze coupled vibrations in curved wells, and the model included nonlinear contact forces and damping effects from drilling fluid. FEA approaches can be computationally inefficient due to requiring a high element count to avoid long, thin elements due to the slender geometric shape of the drill string. It is important to avoid elements with high aspect ratios because of convergence and numerical instability issues.

Based on the current review of drill string dynamic models, there remains a need for fast and accurate vibration simulation models for deviated wells with inclination and azimuth changes. The author’s literature search did not unearth stiff-string simulation models that can accurately predict drill string dynamics within reasonable simulation time while considering the effects of intermittent contact with the deviated wellbore. This paper develops a novel rigid segment deviated drill string dynamic model based on the Newton–Euler formulation using a fast intermittent contact algorithm and a nonlinear stiction/friction model. A case study for well profiles with different inclinations is analyzed, and this work can be extended to variations in both azimuth and inclination without any additional complexity.

### 2.3. Fatigue life determination, damage prediction and damage types

Despite many manufacturing and material improvements, fatigue failure in drill pipes is still an important issue in the drilling industry.^7^ The drill pipe is one of the weakest parts of the drilling rig,^57^ and most of the drill pipe’s failure can be attributed to fatigue damage.^22^

Fatigue is a cumulative and non-reversible phenomenon due to the cyclic loadings applied on the drill pipe, causing propagation of cracks to a critical length, which could cause drill string failure by either wash-out or twist-off. There are some weak points in the drill pipe where the stress concentration is much higher and has a higher tendency to initiate cracks. These stress concentration points can be due to surface irregularities or pipe geometry. Drill pipe surface irregularities can be caused by mishandling the pipe or corrosion pitting, and examples of stress concentration due to pipe geometry are threaded connections and upset areas.^11^

Many types of inspections, such as visual, ultrasonic, electromagnetic and dye penetration tests, can detect cracks and prevent fatigue failure. However, these methods can be time-consuming and have technical limitations in relieving microscopic cracks. Mechanical fatigue calculation based on simulations can be used to reduce the limitations in inspection methods and better determine when the drill pipe should be removed from service.^7^

Fatigue prediction models used in industry are usually based on the recommendations given in API RP7G and ISO 10407, which were developed based on the cumulative fatigue model by Hansford.^58^ Calculating bending stress caused by rotating the drill pipe in a dogleg is a key part of this fatigue model. However, Lubinski^59^ assumes that the curvature of the drill pipe is identical to the dogleg. Sikal et al.^7^ showed that this assumption leads to underestimation of fatigue damage, and the stress distribution of the drill pipe segment depends on its position, wellbore architecture and tortuosity by using case studies. The simulation was carried out using the ABIS^®^ numerical model, which discretized the drill string to very small beam elements and accounts for the external forces applied to the drill string, such as hydraulic forces and temperature effects. The contact between the drill string and the wellbore is calculated using an iterative process. That research work provides great insight into fatigue prognosis in deviated wells; However, this model does not include the effects of drill string vibrations in its calculations and has provided limited details about the model development.

Different types of vibrations cause complex loading and combined stresses in the drill string, and these cyclic loads can cause fatigue failure.^11^ Don and Rideout^60^ developed a fatigue prognosis model and used a multi-axial, non-proportional and variable amplitude (MNV) fatigue model because of the complex load characteristics of the drill string. This model uses a 3D lumped segment model using the Newton–Euler formulation, and the fatigue analysis was carried out using SalomeMeca^TM^ open-source analysis tool. Don and Rideout^60^ research was limited to vertical wells, whereas this paper expands it to deviated wells.

## 3. Methodology

Discretizing the drill string into rigid segments and combining these segments using a compliant joint element to include flexibility has been successfully implemented for vertical and horizontal drill strings. Such a formulation can capture axial, torsional and lateral vibrations and accurately model the drill string’s internal stresses. Rideout et al.^43^ use a multi-segment formulation in BGs to model the dynamics of the horizontal and vertical sections of the drill string. However, that model does not include lateral vibrations in the build section and does not automatically detect wellbore contact. This research aims to improve this formulation by incorporating a novel friction model and a fast contact-calculation algorithm developed specifically for the drill string.

A detailed description of the development of the simulation model is provided in this section. First, the BG formulation is introduced. Next, the rigid body submodel for the drill string, collar and stabilizer segment will be derived, and these segments will be joined together using a compliant joint segment that captures the axial, shear, bending and torsional stiffness of the drill string. In the fourth section, friction models available in the literature are summarized, and a new hybrid friction model is presented. The contact algorithm development is discussed in the fifth section, along with calculations needed for the friction model. The sixth section will describe the top drive boundary conditions and the first drill string segment, which elongates to emulate the drilling progression and installation of new drill pipes. The final section will focus on the bit model, which guides the drill string along the well profile at a specified or calculated penetration rate.

### 3.1. BG formulation

The BG method is a graphical modeling technique that uses the rate of energy transfer as the unifying principle to represent dynamic systems across various domains such as mechanical, electrical, hydraulic and thermal. BGs provide a structured and organized way to represent system dynamics that can be translated into mathematical equations, such as state-space equations, which can then be used for simulation and analysis. Moreover, by focusing on energy flow and power exchange, BGs offer a clear understanding of how energy is stored, dissipated and transformed within a system.^61^

The BG method uses “effort” and “flow” as fundamental power variables to model dynamic systems. Effort represents a generalized intensive variable or driving force (like voltage, force, or pressure), while flow represents a generalized extensive variable or rate of transfer (like current, velocity, or volumetric flow rate). These are power conjugate variables, meaning their product equals power, the rate of energy transfer.

In the BG notation, the half arrows indicate the direction of algebraically positive power flow, and the short causal stroke normal to one end of the bond indicates the input–output structure of the equations of the bonded elements. Consider a bond between two elements, “A” and “B.” A causal stroke adjacent to element “A” indicates that the effort is defined by element “B” and provided to element “A” as input. Meanwhile, element “A” defines the flow variable and provides it to element “B” as an input.

Even though BG formulations were used in developing this model, all the figures and equations in this paper have been converted while preserving the original notation for easier understanding for readers unfamiliar with the BG method. Occasionally, for clarity, the force or moment (generalized effort) and velocity (generalized flow) on some bonds and elements are explicitly noted.

**Figure 1.**
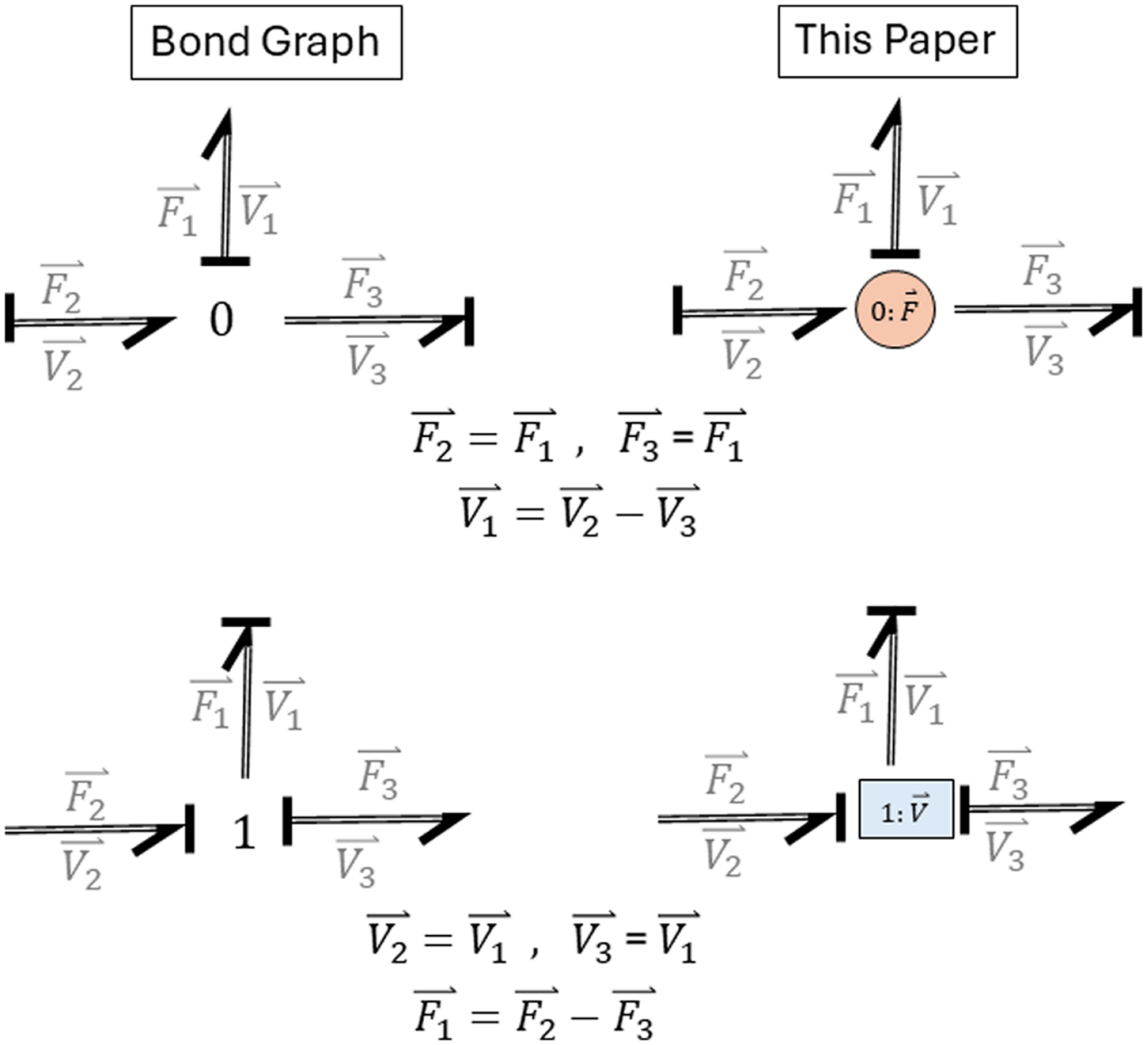
Notation for bond graph junctions.

0-junctions and 1-junctions are essential elements in the BG formulation that connect different parts of a system and enforce Kirchhoff’s energy conservation laws. Figure 1 shows an example of these junctions, and the diagram’s blue boxes and red circles are analogous to “1-junctions” and “0-junctions” in BG notation. The text inside the shapes indicates a common flow or effort at the junction. Kirchhoff’s node law is enforced by a 0-junction, which bonds elements with a common effort (force or moment) and enforces a flow (translational or rotational velocity) summation with algebraic signs determined by the half-arrow directions. Similarly, Kirchhoff’s loop law is enforced by the 1-junction, which bonds elements with a common flow and enforces an effort summation. The causal strokes in Figure 1 indicate the bond that defines the flow, and the equations are written in a manner consistent with those causal strokes.

In BG modeling, energy storage and dissipation elements play crucial roles in representing the dynamic behavior of systems. The green oval shapes in BG schematics in Figures 3, 5, 6, 7 and 10 indicate idealized capacitances, inductances and resistive elements. Storage of energy by accumulation of effort is represented by capacitive (C or K) elements. In mechanical systems, it represents potential energy storage, like a spring storing energy when compressed or stretched. Similarly, the storage of energy by the accumulation of flow is represented by inductive (I, J, or M) elements. In mechanical systems, it represents kinetic energy storage, like a mass storing energy due to its motion. Resistive (R) elements dissipate energy, and these represent friction or damping in mechanical systems.

The purple pentagon shape in BG schematics can either be a source of effort or flow to the system. Gray stadium boxes in the diagram represent transformer elements or gyrator elements. In BG formulation, transformer elements transform effort and flow between bonds with a fixed ratio, and the gyrator elements transform effort to flow and flow to effort. The plaque boxes in the diagrams show mathematical operations or algorithms that provide inputs or outputs for the system. All these elements have descriptive names so that the readers can understand the functionality of each element without any ambiguity.

When developing multibody dynamics (MBD) models, sometimes it is easier to compute velocities and forces in body-fixed coordinates, such as forces on drill segment endpoints ( F→Ai and F→Bi ). Hence, both global and body-fixed coordinate systems were used in this model, and the coordinate frame used in each equation/figure is indicated by the left superscript “0” for the global coordinate system and “i” for the body-fixed/inertial coordinate system.

### 3.2. Rigid body submodel

**Figure 2.**
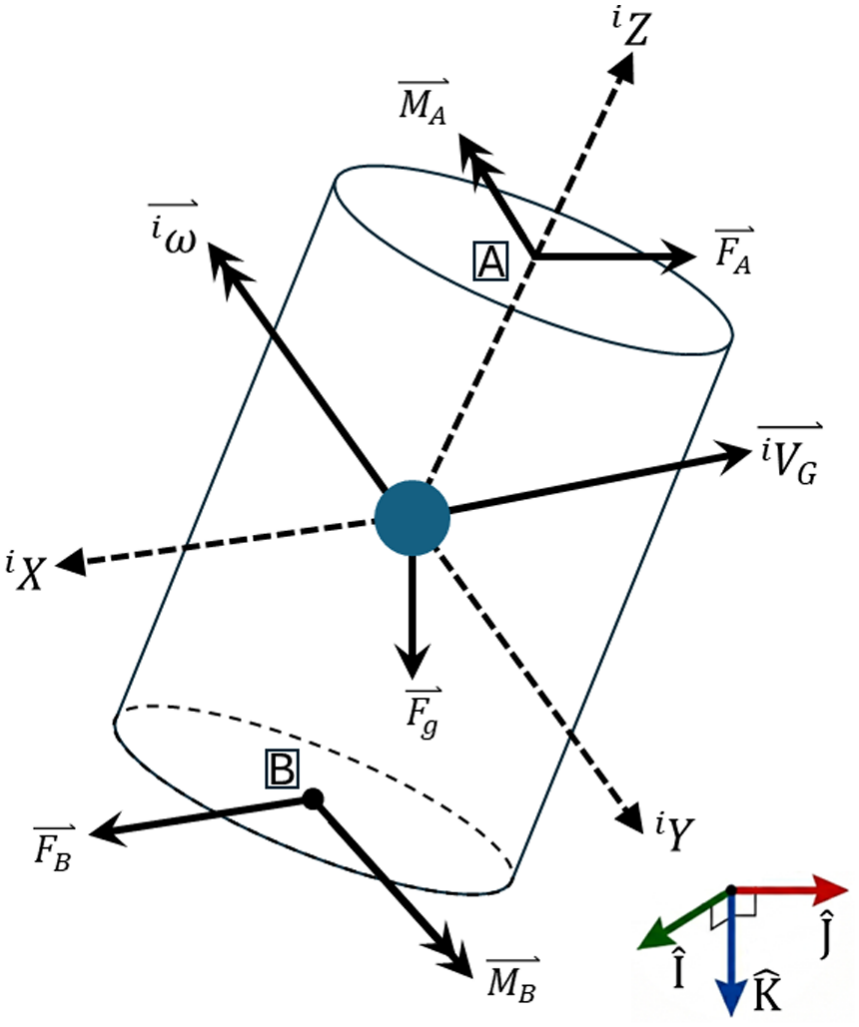
Free-body diagram for a drill string segment.

**Figure 3.**
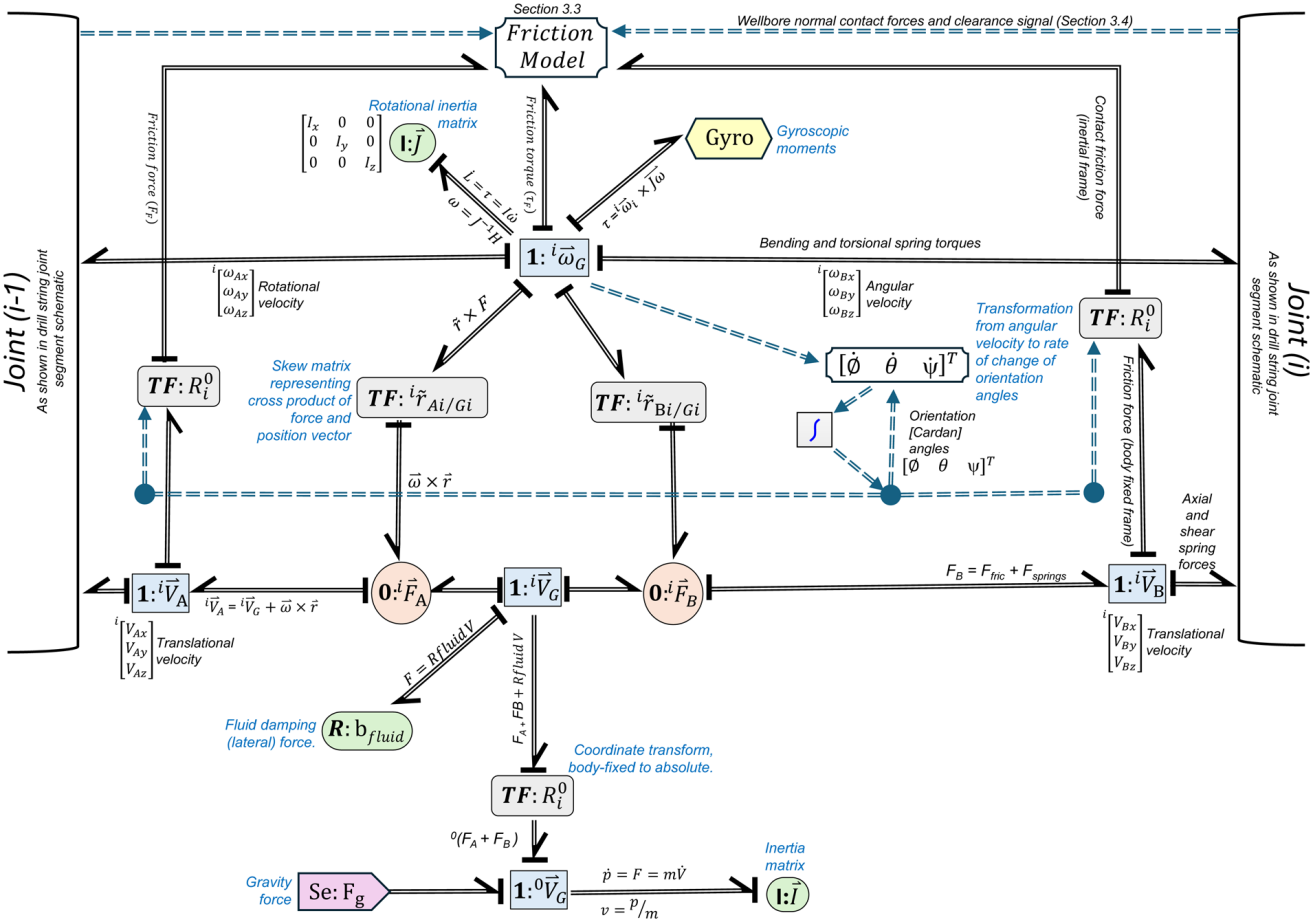
Rigid drill string segment schematic.

Figure 2 shows a free-body diagram of a cylindrical drill pipe segment, with connection points A and B at which the segment is joined to the adjacent one by springs representing axial, shear, bending and torsional internal forces. Friction and wellbore contact forces also act on the planes containing points A and B. The equations below summarize the Newton–Euler formulation for segment i, which is depicted in Figure 3.

(1) F→Ai+F→Bi−FgK^=dp→dt=ddtmV→Gi

(2) =m(V·GXiV·GYiV·GZi)+ω→i×mV→Gi

(3) M→Ai+M→Bi+r→A/Gi×F→Ai+r→B/Gi×F→Bi=dH→dt=ddtJiω→

(4) =J(ω·Xiω·Yiω·Zi)+iω→×Jiω→G

In the above equations, $\vec{p}$ and $\vec{H}$ indicate the linear and angular momentum of the rigid body segment, and $\hat{K}$ represents a unit vector in the global *z*-direction. Linear and rotational inertias of the rigid segment are m and J . The position vector from G to A is given by r→A/Gi which operates on the force to create moment.

In Figure 3, the center of gravity velocity is transformed to an inertial frame with the rotation matrix $R_{i}^{0}$ , which is a function of Cardan orientation angles.^62^ The Cardan angle derivatives are functions of the body-fixed angular velocity components and the angles, implemented with the block diagram structure depicted. The cross-products that define moments due to forces can be expressed as the product of a relative position vector skew matrix ( ${\tilde{r}}_{A/G}$ ) and the force vector:

(5) $$\vec{M}={\tilde{r}}_{A/G}\vec{F}$$

The same relative position vector participates in a cross-product defining relative velocity, which is added to the center of mass velocity to give the velocity of “hinge points A and B,” where springs are connected, and friction and contact forces are applied.

(6) $${\vec{V}}_{A}=\vec{V_{G}}+\vec{\omega }\times {\vec{r}}_{A/G}=\vec{V_{G}}+\left(-\right){\tilde{r}}_{A/G}\vec{\omega }$$

The “ ${\vec{r}}_{\mathrm{Ai}/\mathrm{Gi}}$ ” blocks in Figure 3 represent power-conserving transformations that operate on force to create moment and on angular velocity to create relative linear velocity, using the same position vector as a parameter. The causal stroke adjacent to the block indicates a relative velocity flow definition. The relative velocity is an input to the FAi force node that does the velocity summation in the relative velocity equation. The upper bond with the causal stroke not adjacent to the ${\vec{r}}_{\mathrm{Ai}/\mathrm{Gi}}$ block shows that an input force to the block is converted to a moment, one of several that are applied to the body at its angular velocity node ωi .

The friction force model in Section 3.6 accepts as inputs the cylinder endpoint velocity $v_{A}$ or $v_{B}$ transformed to the inertial frame, the angular velocity of the cylinder, and wellbore contact forces calculated in Section 3.8.

### 3.3. Compliant joint between segments

**Figure 4.**
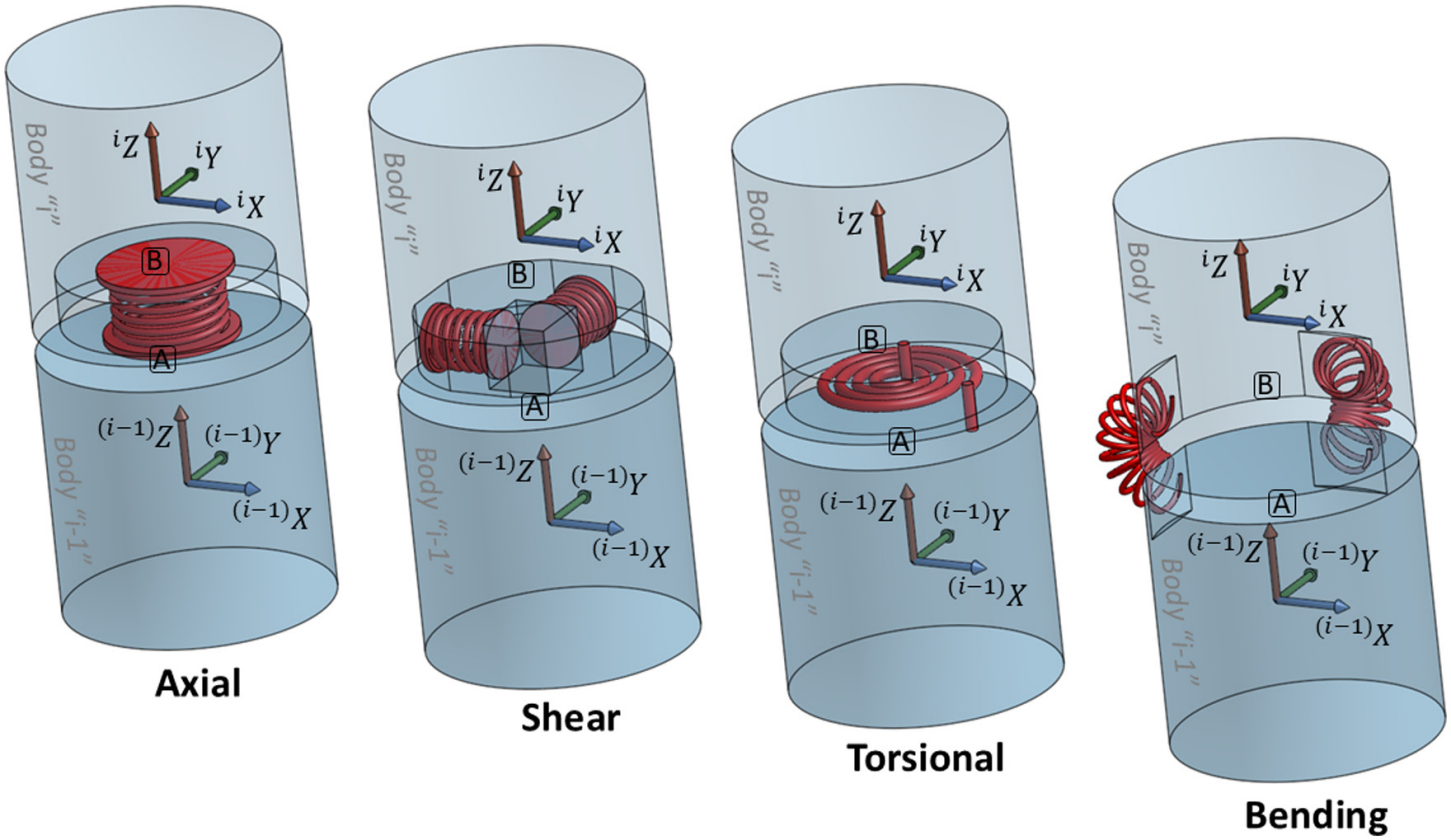
Axial, torsional, shear and bending springs between segments.^63^

Moments $M_{A}$ and $M_{B}$ are imposed by connecting springs between adjacent elements, as are shear and axial force components of $F_{B}$ and $F_{B}$ , according to the stiffnesses and constitutive laws below and connected as in Figure 4.

(7) $$K_{\mathrm{tors}}=\frac{\mathrm{GJ}}{\Delta z}$$

(8) $$K_{\mathrm{bend}}=\frac{\mathrm{EI}}{\Delta z}$$

(9) $$K_{\mathrm{axial}}=\frac{\mathrm{EA}}{\Delta z}$$

(10) $$K_{\mathrm{shear}}=\kappa \frac{\mathrm{AG}}{\Delta z}$$

The length of each rigid drill string segment is denoted by Δz , and the cross-sectional area is denoted by *A* in the above equations. *I* and *J* are the area moment and polar moment of area, and *E* and *G* are elastic modulus and shear modulus, respectively. The non-uniform shear behavior due to the hollow drill string cross-sectional geometries is accounted for by the Timoshenko shear coefficient ( κ ).

**Figure 5.**
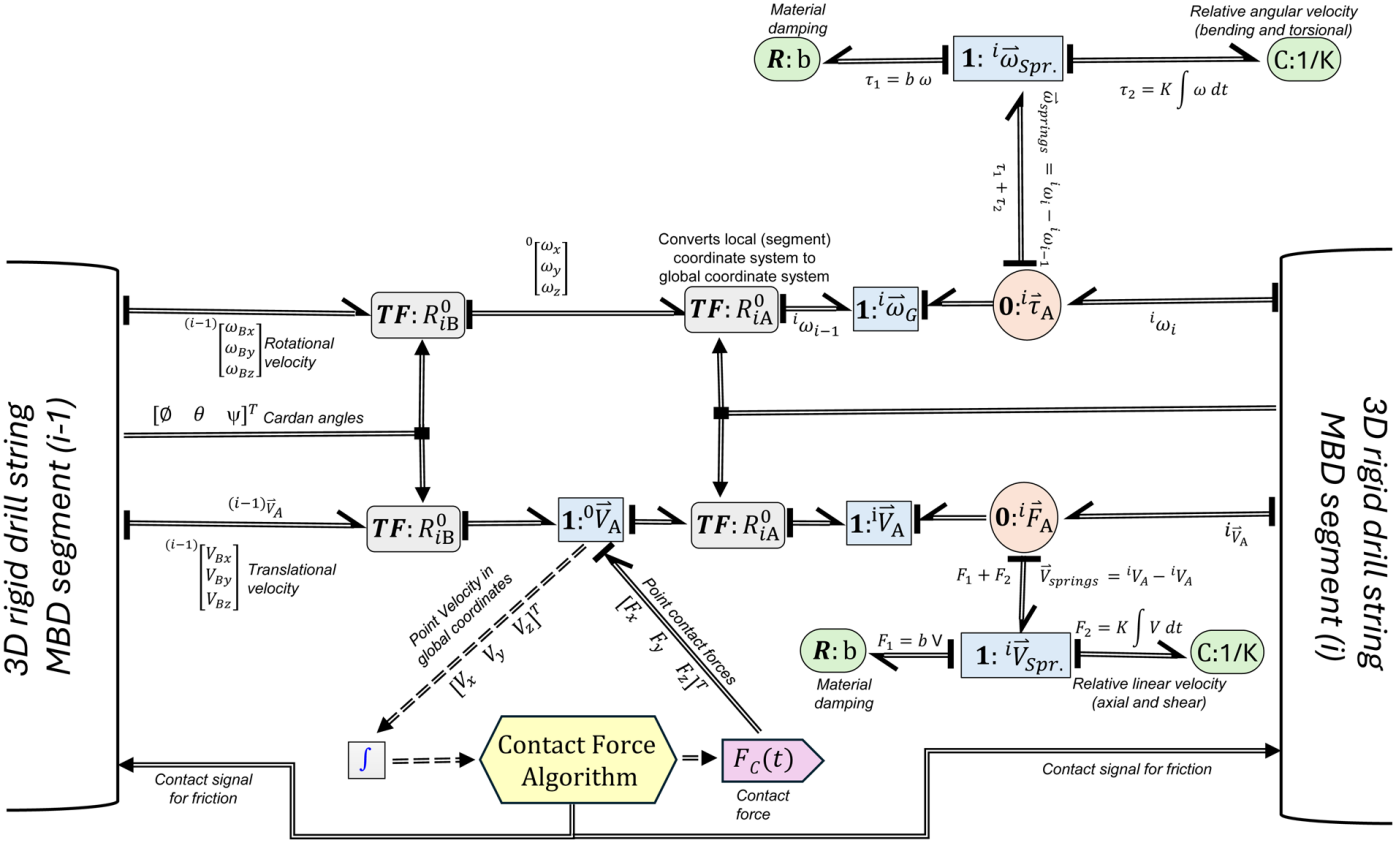
Joint segment BG schematic.

String flexibility was modeled inside the BG joint segment as shown in Figure 5 by implementing the below given equations:

(11) [FshearXFshearYFaxial]=[Ksh000Ksh000Kax][∫V→Bi−V→Aidt]

(12) [MbendingXMbendingYMtorsional]=[Kbend000Kbend000Ktors][∫ω→Bi−ω→Aidt]

where ω→Ai and V→Ai are transformed from the body-fixed coordinates of the “i-1 element” to global coordinates and then transferred to the “i” body-fixed coordinates.

(13) V→Ai=[R0i][Ri−10][i−1VA→]

(14) ω→Ai=[R0i][Ri−10][i−1ω→A]

After the coordinate transfer, both A velocity in the “i element” and the B velocity in the “i-1” element are represented in the same coordinate system, so that relative movement between rigid segments can be compared for modeling spring and damping elements in axial, torsional and transversal directions as shown in Figure 5.

### 3.4. Top boundary condition and first rigid body

**Figure 6.**
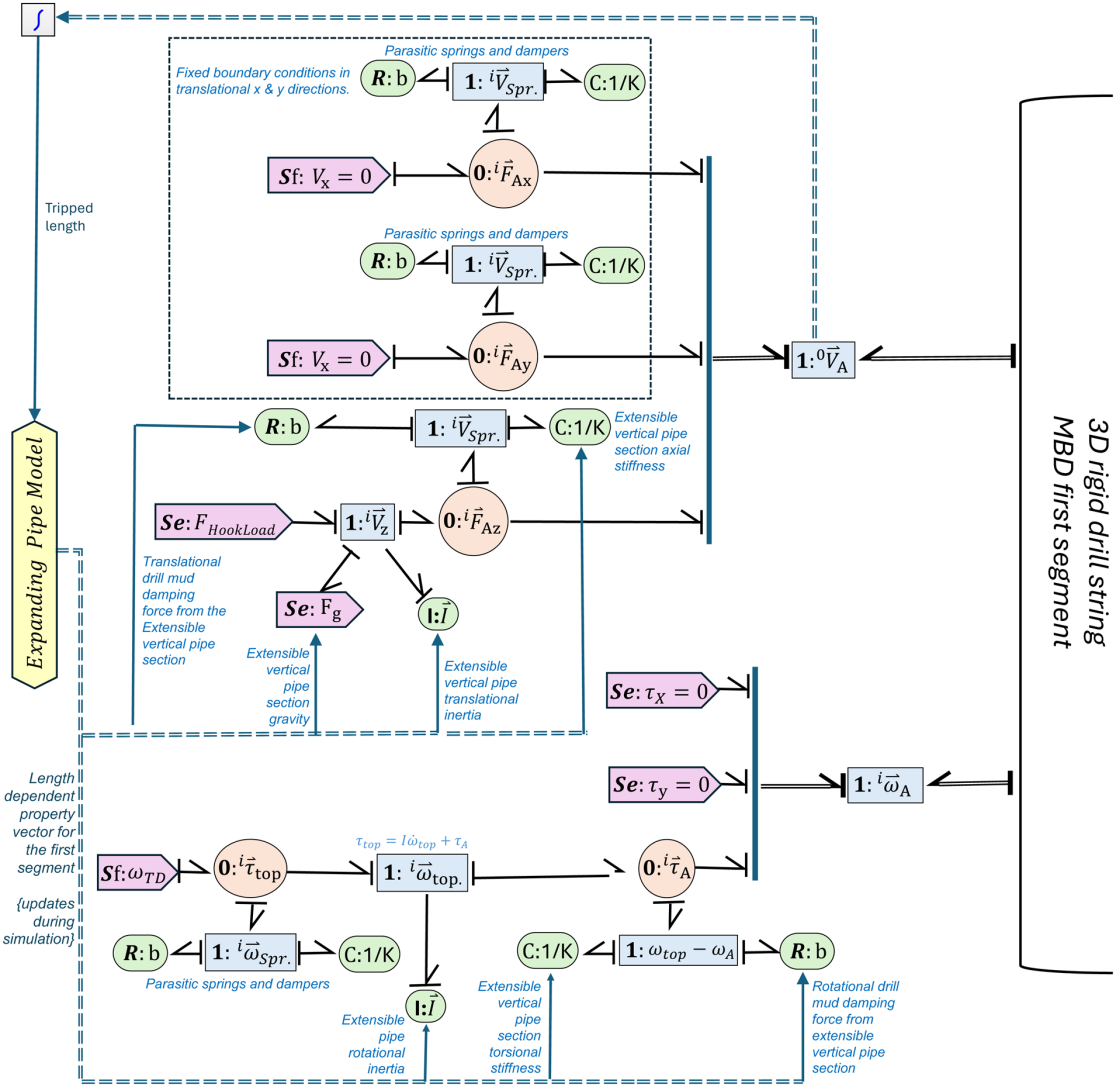
First joint segment schematic.

Figure 6 depicts the first joint element, which includes elements to simulate the first rigid drill string segment, top drive and hook load. The first drill string segment differs from the rest of the drill string segments since the length of the first segment continually expands as the string grows with the progression of drilling, enabling an unbroken connection between the top drive and the rest of the drill string. This continually updating string segment enables simulation of drilling progress and tripping operations.

The top boundary condition in this simulation model’s *x* and *y* translational direction was constrained using two zero velocity flow elements, which prevents lateral motion, emulating the connection between the topdrive and the drill string. A parasitic damper and parasitic spring with high stiffness were included to avoid causality conflicts. Similarly, rotational freedom about the *x* and *y* axes in the top boundary conditions was also constrained using zero velocity flow elements.

The *z* rotational direction top boundary condition was constrained using the rotational velocity function to emulate the string rotation speed imposed by the top drive. The *z* translation direction top boundary has a translational force equal to the hook load. Since the first drill string segment expands as drilling progresses, the top boundary point on the simulation model’s *z* translational direction remains stationary throughout the simulation.

The topmost vertical segment is an expandable two-dimensional (2D) inertia element with axial stiffness, torsional stiffness, gravitational forces and drilling mud-damping effects. Lateral dynamics in this segment are not included because the drill pipe in the initial vertical section is under tension, limiting lateral movement, and the clearance between the well prevents contact whirl. However, if required, the effects of lateral vibrations in the vertical section can be implemented by dividing the first segment into two or more elements, increasing the length as the tripping operation progresses to capture lateral vibrations.

The value for a unit length of the drill string for each of these parameters was calculated based on mechanical properties. During the simulation, the “Tripped Drill String Calc” algorithm multiplies these values by the length of the expandable first drill string, which is equal to its initial length plus the tripped length. Afterward, these values are applied as input signals to variable stiffness, inertia, or force elements, as shown in Figure 6.

### 3.5. End boundary condition and bit forces

**Figure 7.**
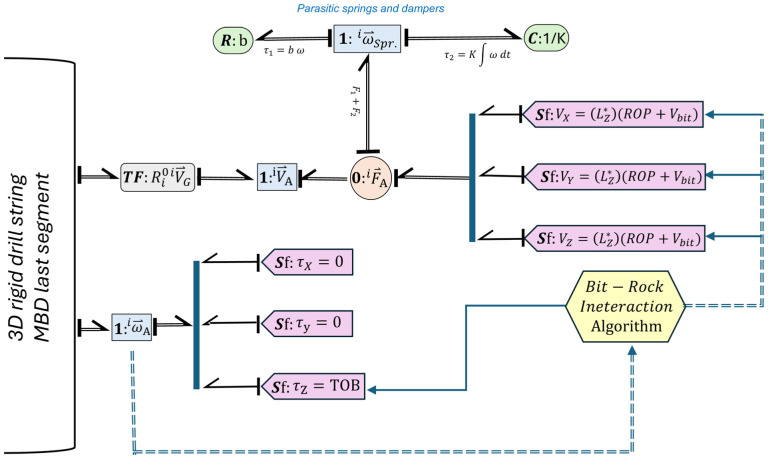
End joint segment schematic.

The end joint element shown in Figure 7 has translational boundary conditions that control the rate-of-penetration model and guide the bit along the deviated well path. The rotational component in the *x* and *y* directions has free boundary conditions, and in the *z*-direction, torque-on-bit (TOB) and bit-rock interaction forces are applied.

Bit-rock interaction provides coupling between the axial and torsional dynamics and generates vibrational excitation due to the destructive force of drilling. Moreover, the bit-rock interaction also dictates the TOB, WOB and ROP. Teale^64^ introduced the concept of mechanical specific energy (MSE)—the energy required to remove a unit volume of rock, which provided a direct link between TOB, WOB, RPM and ROP. Warren and Sinor^65^ developed an ROP model specifically for PDC bits that accounts for rock strength, bit geometry and the cleaning effect of drilling mud. Detournay and Defourny^66^ introduced a phenomenological model which characterized the interaction between the rock and PDC bits by coexistence of rock cutting and frictional contact and further developed in Detournay et al.^67^ by introducing two new quantities, contact strength and length to better model the frictional characteristics.

Yigit and Christoforou^24^ developed a computationally efficient empirical bit-rock interaction model that calculates the ROP and TOB, which facilitated the coupling of axial and torsional dynamics of the drill string. This model was improved by Sarker et al.^68^ by introducing a threshold WOB and bit rotational speed, below which the ROP would equal zero, and the TOB will be calculated based on the frictional force. Sarker et al.^44^ further improved this model by allowing the bit to move in the horizontal direction, and this research work further improves that bit-rock model by enabling the bit to move in any direction along the specified deviated well profile. For a detailed description of the equations used in calculating the ROP and TOB, the reader should refer to Yigit and Christoforou^24^ and Sarker et al.^68^

As shown in Figure 7, the calculated TOB is applied to the endpoint of the last drill segment as a torque vector in the *z*-direction in the body-fixed coordinates of the bit. The velocity of the last drill string segment endpoint was applied in the global direction and has two components: ROP and the sinusoidally lobed profile function that imparts an axial velocity boundary condition based on the assumed contact surface between the PDC bit and the rock to emulate bit vibrational velocity ( $V_{\mathrm{bit}}$ ).

Modeling vibrations generated by rock fracture at the bit is complex and depends on many parameters, such as rock type, bit type and WOB. Therefore, many research works, including this paper, have used sinusoidal functions to emulate bit vibrations. These functions will depend on the expected average vibrational amplitude and number of bit lobes. This simplified sinusoidal assumption can be replaced by an article published by the authors of this paper,^69^ which provides a bit-rock force time series using an Artificial Neural Network (ANN) approach based on WOB and ROP.

The ROP in this model can either be specified by the user or calculated using equations derived by Yigit and Christoforou^24^ and Sarker et al.^68^ Based on the ROP, the bit follows a user-defined well profile when simulating drilling progression and tripping operations. As shown in Figure 7, bit velocity is implemented using velocity functions in all three translational directions.

### 3.6. Friction model development

Friction is a complex phenomenon affecting the drilling operation’s dynamics and efficiency. In deviated wells, the contact between the wellbore and drill string creates frictional torque during rotations and frictional drag during tripping operations. These frictional forces can cause wear in drilling tools and, in some severe cases, shearing of drill pipes. Moreover, the frictional force on the drill string can create vibration, especially stick-slip.

**Figure 8.**
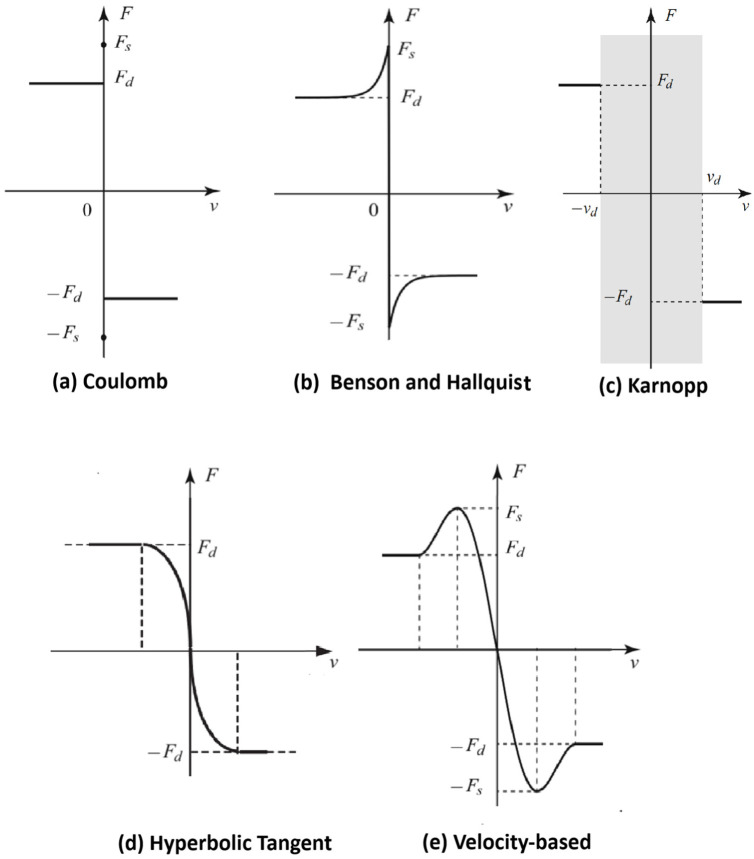
Few of the friction models available in the literature.^70^ (a) Coulomb, (b) Benson and Hallquist, (c) Karnopp, (d) hyperbolic tangent and (e) velocity-based.

A Coulomb friction model of this effect is shown in Figure 8(a) and is difficult to implement in simulation despite appearing mathematically simple due to its discontinuity. Equation (15) expresses Coulomb friction in terms of relative velocity *v* between two rigid bodies.

(15) $$F\;\left\{ \begin{array}{cc} \leq \mu_{s}F_{n} & v=0\\ =\left(-\right)\mu_{d}F_{d}\mathrm{sgn}\left(v\right) & v\neq 0 \end{array}\right.$$

Stick-slip happens due to the difference in static and dynamic frictional coefficients. It occurs when the relative sliding velocity between two surfaces approaches zero, requiring a force larger than the dynamic frictional force to break the static condition. Moreover, experimental frictional force analysis has shown that the friction force varies with relative velocity during the region leading up to zero velocity. However, these effects are not included in the Coulomb friction model equations. The Benson and Hallquist^71^ friction model shown in Figure 8(b) accounts for the difference in static and dynamic frictional coefficients and can be expressed analytically using the equations below.

(16) $$F=F_{d}-\left(F_{s}-F_{d}\right)e^{-c\left|v\right|}\mathrm{sgn}\left(v\right)$$

The sign function and the absolute value in the exponential in the above equation cause nonlinear behavior, so simulating accurate friction is challenging and can cause model instabilities. In addition, at zero velocity, this model inaccurately computes friction force as zero. However, this is not significant because the drill pipe operates with some rotational velocity during most simulation time. Furthermore, to simulate true zero velocity sticking, the frictional force must be equal and opposite to the driving force on the object, which requires switching between equation models.

Karnopp^62^ proposed a straightforward method to model friction with true zero velocity stiction without equation reformulation or introducing numerical stiffness problems. This model has two distinct phases: the stick phase, which covers a small region when velocity reaches zero, and the slip phase, which covers the sliding velocities of the object as shown in Figure 8(c). The main drawback of the Karnopp friction model is that it requires information about the resultant of the external forces applied to the object. In mechanical systems with multiple contacts per body, evaluating this force can be difficult.^70^

Margolis^72^ developed a self-contained stick-slip frictional element based on the Karnopp model to eliminate the abovementioned drawback. This element requires velocity input from the attached system and outputs a frictional force based on the self-contained test to identify stick and slip phases without requiring additional information from the attached system. In the stick phase, the frictional force is produced by a small but finite displacement of a highly stiff spring-damper system, enabling true zero velocity stiction behavior. However, having different operational phases can introduce computational burden, slowing the overall simulation speed in complex models.

In all the abovementioned friction models, a discontinuity at a zero velocity point can cause simulation instabilities and computational burden. To mitigate this issue, continuous equations have been developed for the frictional forces using mathematical expressions such as the hyperbolic tangent function. Mostaghel^73^ formulated the function in equation (17) and illustrated in 8(d).

(17) $$F=\left(-\right)F_{d}\;\tanh\left(v/v_{d}\right)$$

The hyperbolic tangent friction model does not include the difference between the static and dynamic frictional coefficients. Hence, this model cannot be used to model the stick-slip conditions. To incorporate static friction, some models have introduced additional curves near zero velocity intervals. This piecewise assembly of curves to generate the frictional equation adds simulation complexity.^70^

Wang and Rui^74^ developed velocity-based friction models based on trigonometric functions (equation 18), depicted in Figure 8(e). In this model, C, B and E are parameters that must be adjusted to match the shape of the required frictional coefficients, which adds additional complications when implementing this model.

(18) $$F=\left(-\right)F_{s}\sin[{\mathrm{Ctan}}^{-1}\left(B.v\right)-E\{\left(\mathrm{Bv}\right)-\tan^{-1}\left(\mathrm{Bv}\right)\}]$$

To efficiently simulate friction with static and dynamic friction coefficient, the authors of this paper have combined hyperbolic tangent and exponential functions into a new friction model, expressed in the following equations.

(19) $$F=\left(-\right)\mu \times N\times \tanh\left(\frac{\alpha v}{v_{t}}\right)$$

(20) $$\mu =\mu_{k}+\left(\mu_{s}-\mu_{k}\right)*e^{-\lambda \left|v\right|}$$

**Figure 9.**
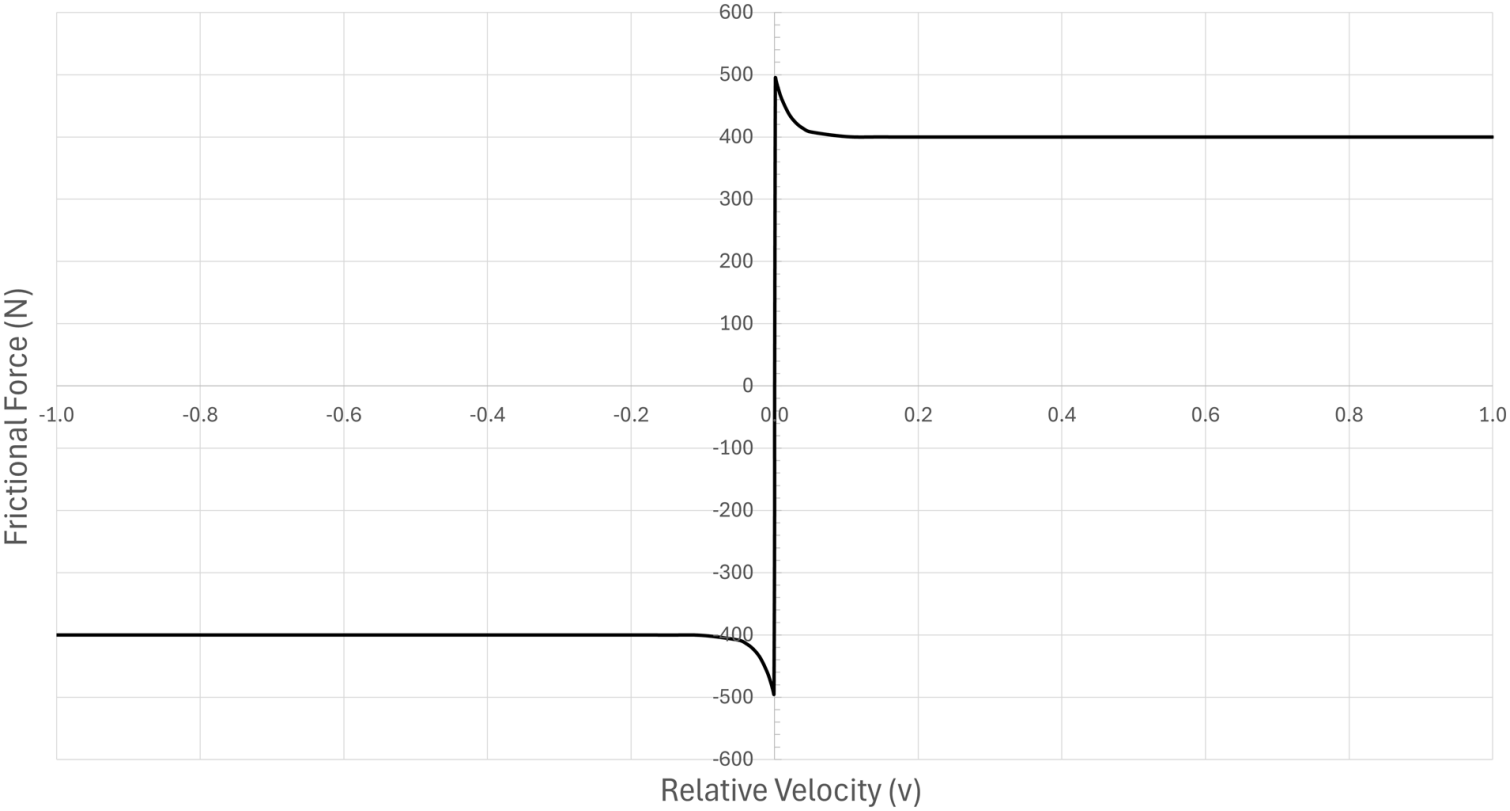
Sample graph of the new friction model.

In this combined tanh-exponential friction model, λ,α and $v_{t}$ are model coefficients and given below are how these parameters affect the shape of the curve:

For most applications $\lambda =50,\alpha =5\;\mathrm{and}\;v_{t}=0.001$ would provide accurate results and users do not need to tune these parameters for each implementation. Figure 9 depicts a sample model where $N=1000N,\mu_{k}=0.4\;\mathrm{and}\;\mu_{s}=0.5$ and shows that the tanh-exponential friction model behaves as expected.

**Table 1.** Parameters and shape of the friction graph.

Para.	Effect on the shape of the curve
λ	Transition steepness between $\mu_{s}$ and $\mu_{d}$
α	Increasing will reduce the error in $\mu_{s}$
$v_{t}$	Controls how fast the velocity affects the friction

A limitation of this model is that the maximum value of the frictional force given by the equation is slightly less than the static frictional force and occurs slightly above and below the zero velocity value. This happens because this is a continuous equation, and at a velocity equal to zero, the frictional force will also equal zero. Hence, this does not exhibit true zero velocity stiction behavior. Yet, the model imitates stiction behavior by rapidly switching between positive and negative velocities by a very small value.

### 3.7. Friction model implementation

**Figure 10.**
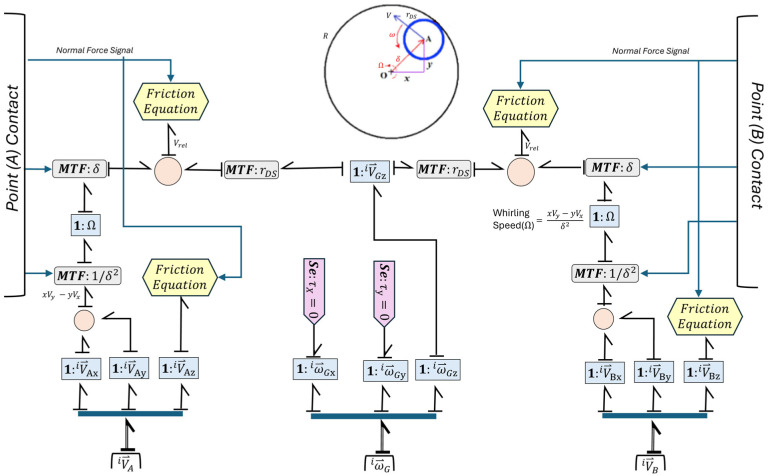
Friction model schematic (this is inside of drill string segment).

Implementation of the friction model is shown in Figure 10. The friction model is placed inside each rigid drill string segment model and generates forces that are applied to the center of gravity (G) and the end plane points (A and B), as shown in Figure 2.

By observing the geometry of the drill pipe and wellbore, Sarker et al.^44^ have implemented the following equation for whirling speed (Ω) . In this equation, δ is the distance between the drill string and wellbore centers, and *x* and *y* values are its directional components.

(21) $$\Omega =\frac{V}{\delta }=\frac{V_{y}x-V_{x}y}{\delta^{2}}$$

The relative velocity between whirling speed and drill string rotational speed is calculated using the following equation. In this equation, $r_{\mathrm{DS}}$ is the radius of the drill string.

(22) $$V_{\mathrm{rel}}=\Omega \delta +\omega_{Z}r_{\mathrm{DS}}$$

The model computes the relative velocity between sliding surfaces and generates rotational and translational frictional components based on the normal contact force. Equations (21) and (22), used to calculate the velocities, are applicable for rolling with and without slip conditions and enforce a rolling-without-slip constraint as the rotational velocity ( $\omega_{z}$ ) approaches zero.

The above frictional equation does not account for the translational frictional component along the drill string. This component influences the WOB as drilling progresses and affects axial stresses during tripping operations. To implement the tripping frictional force (drag) component, another frictional element, as shown in Figure 10, was included based on the translational velocity along the drill string direction.

### 3.8. Fast point in cylinder contact algorithm

In highly deviated wells, the drill string can be in contact with the top or bottom surface of the wellbore based on the curvature and the tension. These intermittent contacts can affect the drill sting dynamics and boundary conditions. According to McSpadden and Newman,^75^ ignoring the tubular stiffness and assuming continuous contact with the bottom wellbore wall can result in significant errors for wells with dogleg severity (DLS) beyond 30º/100 ft or for stiff tubular sections such as drill collars.

Hence, to model the torque and drag of highly deviated wells, the authors of this paper expanded the “fast point in the cylinder” algorithm developed to model the contact of arms and legs in computer games by James.^76^ This modified contact algorithm, along with validation using torque measurement from a completed deviated well, was published in Liyanarachchi and Rideout.^63^

These modifications include a method to define a wellbore profile using cylinders, a looping algorithm to calculate contact between drill string segments and wellbore cylinders, an overlap calculation to enable a smooth transition between wellbore cylinders and an algorithm to calculate the direction of contact force.

**Figure 11.**
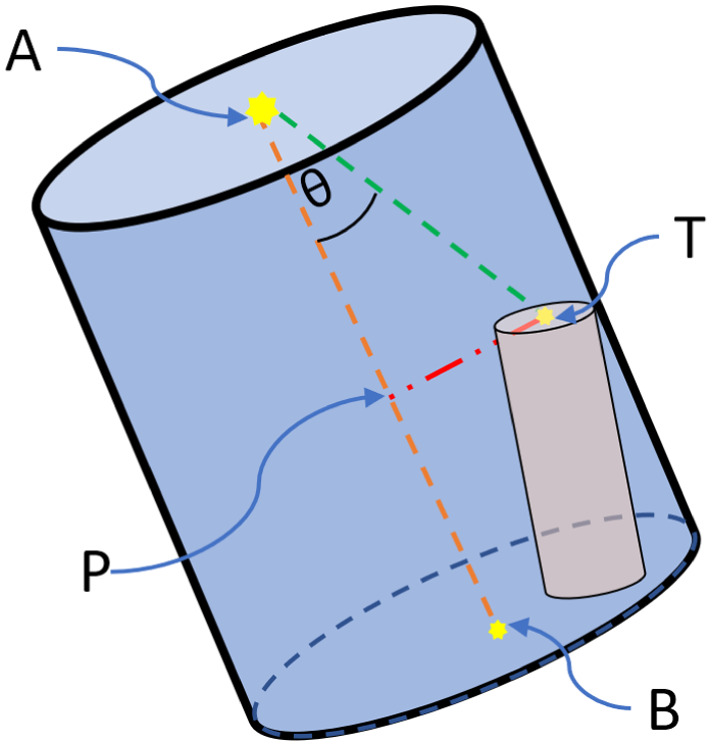
Wellbore cylinder and drill string segment point for contact detention.^63^

In this algorithm, the wellbore was divided into straight cylinder sections defined using the diameter and the top and bottom center endpoints. As shown in Figure 11, the location of drill string segments is calculated by integrating the velocity of the joints in global coordinates. This calculated location information is then fed into the fast contact drill string (FCDS) model to calculate the contact forces and direction, which are then applied to the same junction point using a force function. Moreover, the FCDS model also provides normal forces and the distance between the drill string and wellbore center points for the friction calculations.

This algorithm first selects the top wellbore cylinder and defines two vectors, as shown in Figure 11. The first vector (AB) is between the start and end points of the wellbore cylinder, and the second vector (AT) is between the wellbore start point and the drill string point. Then, the dot product (L) between those two vectors is taken, and if the value is greater than the length of the wellbore cylinder squared $Q^{2}$ , the algorithm will then move to the next wellbore cylinder. This computation step will loop until the wellbore cylinder where the drill string point is located is determined. After that, a perpendicular vector (PT) between the wellbore centerline vector and the drill string point is defined, and its length can be calculated.

(23) $${\left|\vec{\mathrm{PT}}\right|}^{2}={\left|\vec{\mathrm{AT}}\right|}^{2}\left(1-\frac{Q^{2}}{{\left|\vec{\mathrm{AB}}\right|}^{2}{\left|\vec{\mathrm{AT}}\right|}^{2}}\right)$$

Whether or not contact has occurred and, if so, the penetration distance (D) of the drill string point into the wellbore can be calculated using,

(24) $$D=\left(r_{\mathrm{bh}}-r_{\mathrm{ds}}\right)-\left|\vec{\mathrm{PT}}\right|$$

In the above equation, $r_{\mathrm{bh}}$ is the radius of the wellbore and $r_{\mathrm{ds}}$ is the radius of the drill string. If the value of the above equation is positive, then the contact has occurred, and the contact force can be calculated by multiplying the penetration distance with the wellbore-string contact stiffness coefficient.

Next, the contact force direction, which will be equal to the vector PT, can be calculated using the following equation,

(25) $$\vec{\mathrm{PT}}=\left(\vec{A}-\lambda \left(\vec{A}-\vec{B}\right)\right)-\vec{T}$$

(26) $$\lambda =\frac{\left(\vec{A}-\vec{B}\right).\left(\vec{A}-\vec{T}\right)}{\left(\vec{A}-\vec{B}\right).\left(\vec{A}-\vec{B}\right)}$$

**Figure 12.**
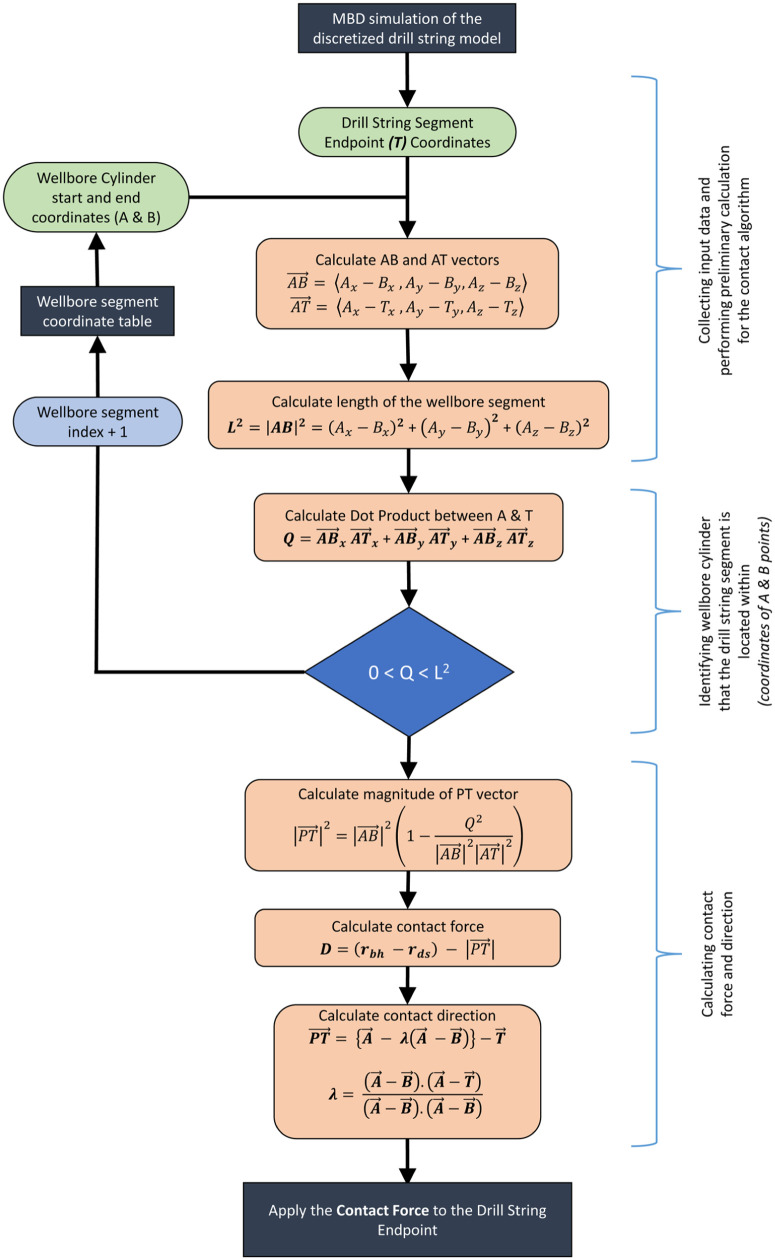
Contact algorithm.^63^

The above paragraphs and Figure 12 summarize the drill string contact algorithm, and readers should refer to Liyanarachchi and Rideout^63^ for detailed derivations, explanations and limitations.

## 4. Case studies and model utilization

The following case studies show the functionality of the contact algorithm and friction model, as well as how the model can be utilized to predict stress history and fatigue life of drilling components. 20Sim^TM^ software package was selected to implement the derived BG model of the drill string and all the algorithms, including the contact model. 20Sim^TM^ has multiple implicit and explicit integrators and supports easy integration of customized equations with BG models, making it an ideal tool for this implementation. In addition to 20Sim, there are many open-source and commercial alternatives such as Simbus, BondGraphTools^77^ and MTT,^78^ that can be utilized to implement the deviated well drill string model.

### 4.1. Case study—whirling vibrations in horizontal sections

**Figure 13.**
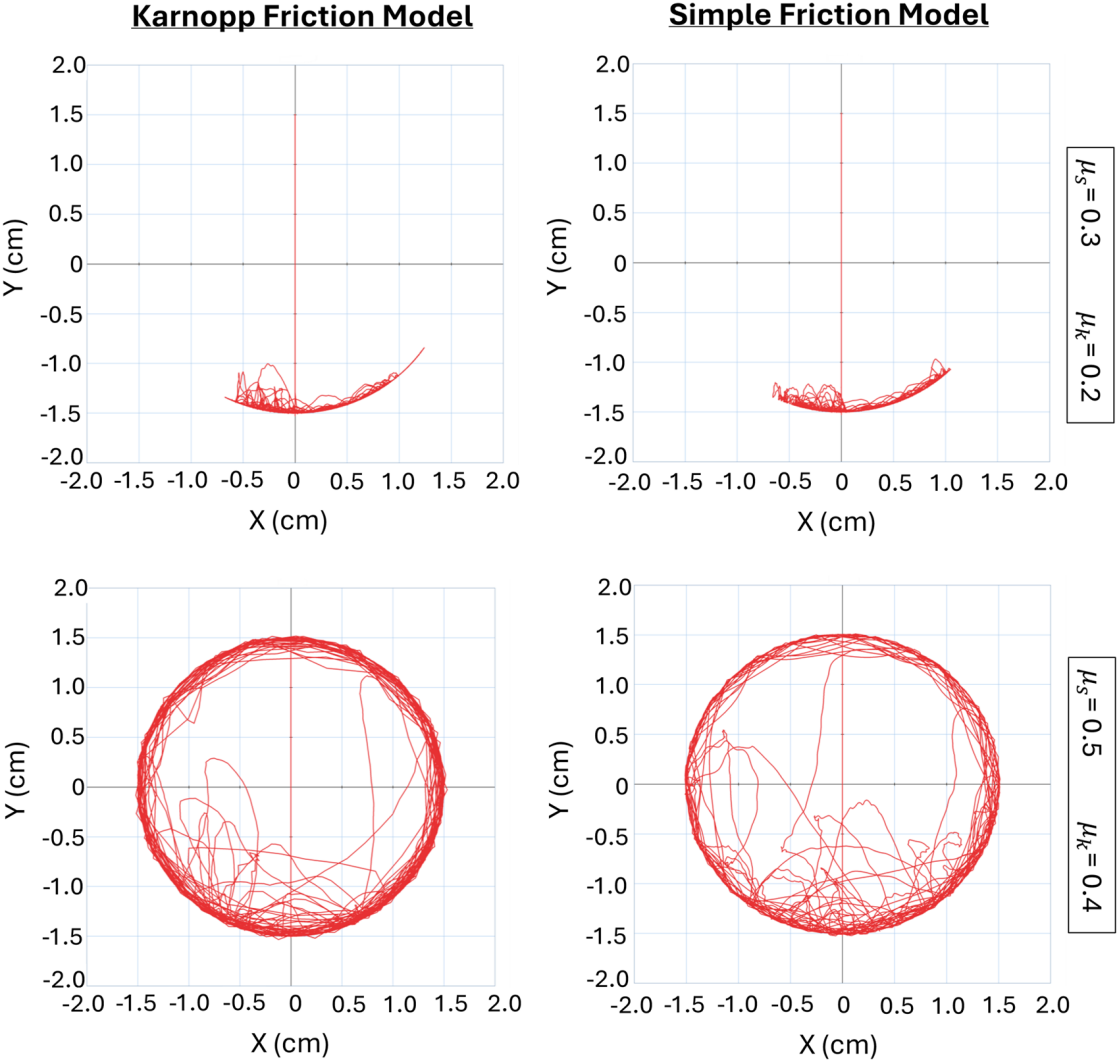
Whirling vibration comparison between different friction models and values.

A 5 m pipe section was modeled with five rigid segments and free boundary conditions. The shaft material was selected as steel, and the wellbore was modeled as horizontal. A constant torque was applied at the top of the first segment to simulate drill string rotations and excite whirling vibrations. This simulation was carried out using the Karnopp, and hybrid tanh-exponential friction models developed in Section 3.6. The analysis results are given in Figure 13.

For low friction, the torque applied to the end of the pipe causes it to climb the circular wellbore until required friction force exceeds the maximum static value, at which point the pipe slides down the wall. For the higher friction coefficients, the combination of pipe inertia and friction allows the pipe to maintain continuous contact with the wellbore, resulting in backward whirl.

Compared with the Karnopp model, which is a true stiction friction model with static frictional force generated at zero relative velocity, the simpler tanh-exponential model captures the same expected behavior with less computational burden, completing the simulation in 18 min instead of 47 min. Backwards differential formulation (BDF) implicit integration method was selected due to its superior stability for stiff simulation systems and adaptive time-stepping. Absolute and relative error tolerances in both models were set to 1e-5, which was selected after a few iterations to ensure that tighter tolerances do not change the results. The time step is selected by the integrator based on stability, error tolerance and convergence criteria. The maximum time step size was constrained to 0.001 s to ensure the integrator would not take time steps that are too large and fail to capture high-frequency oscillations or sudden switching.

The authors found that when the number of string segments exceeds 30, the computational requirement becomes critical for generating results within a reasonable time. This was apparent in the next case study, in which the model expanded to a full drill string comprising 120 segments.

### 4.2. Case study—vibrational characteristics and fatigue analysis of different well profiles

**Figure 14.**
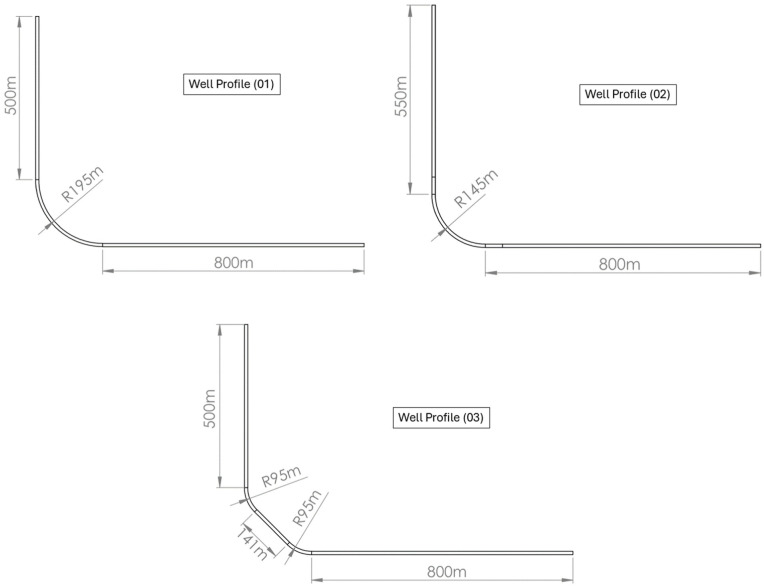
Well profiles used in the case study.

Analyzing the effects of the curvature of the wellbore profile on vibrations and component stresses is essential for safe and efficient drilling. Therefore, as a case study, three different well profiles with the same starting and endpoints were analyzed, as shown in Figure 14. This analysis will show the effects of different dogleg severities and kick-off points on the drill string’s vibration characteristics and fatigue life and how this model can be used as a design tool to select the most suitable well profile during the design stage. The goal is to efficiently predict 3D vibrations throughout the entire drill string without making limiting assumptions about the nature of contact in the build section.

The model has 100 rigid drill pipe segments and 20 rigid drill collar segments. The number of elements was decided based on the previous iteration for developing a similar rigid segment drill string simulation model^63^ and provides a good balance between computational efficiency without compromising simulation results. The dimensions and material properties of rigid segments are summarized in Table 2.

**Table 2.** Drill pipe and collar simulation parameters.

Parameter	Pipe	Collar
Number of elements	100	20
Length of an element	4 m	4 m
Inner diameter (ID)	85 mm	65 mm
Outer diameter (OD)	102 mm	158 mm
Material	Steel	Steel

Drilling is a complex operation that spans multiple days; while it would be feasible to do a full-duration simulation with the proposed model, this paper uses a higher ROP of 0.5 m/s to reduce simulation time to hours instead of days. This case study is intended to demonstrate how stress data representative of the drilling operation, including making the curved section, can be gathered for any time window of interest to the analyst. Another approach to modeling long drilling time would be to take samples from various well sections and apply those stresses over a long time period for fatigue simulation as in the case study in Section 4.1.

In this analysis, the rotational speed was set to 150 r/mim, and the hook load was set so that the transition between tension and compression happens at the beginning of the drill collar section. The simulation begins when the bit is near the deviated well section and ends after 800 s of simulation time, which includes the initial vertical section, completion of the deviated section and a couple of hundred meters of the horizontal well section. The total length of the drill string at the beginning of the simulation is 480 m, and by the end time, the total length is 880 m due to the lengthening of the first rigid segment to account for the progression of the drilling. The simulation took around 38 h using the BDF integrator with tolerances set to 1e-5 to run all three profiles simultaneously.

Throughout the 800-s simulation window, the drill string was subjected to bit bounce and whirling vibrations. Moreover, the high ROP value also induced high axial, bending and shear stresses on the drill string. This simulation window represents a harsh operational condition, equal to weeks of a drilling operation. Moreover, whenever drilling vibrations are observed in a typical drilling operation, the rig operator would take action to mitigate vibrations. Therefore, harsh drill string vibrations only last a few minutes, unlike in this simulation. However, the following operational conditions were selected as the worst-case vibrational scenario to analyze fatigue damage at each point of the well, especially at the build section.

**Figure 15.**
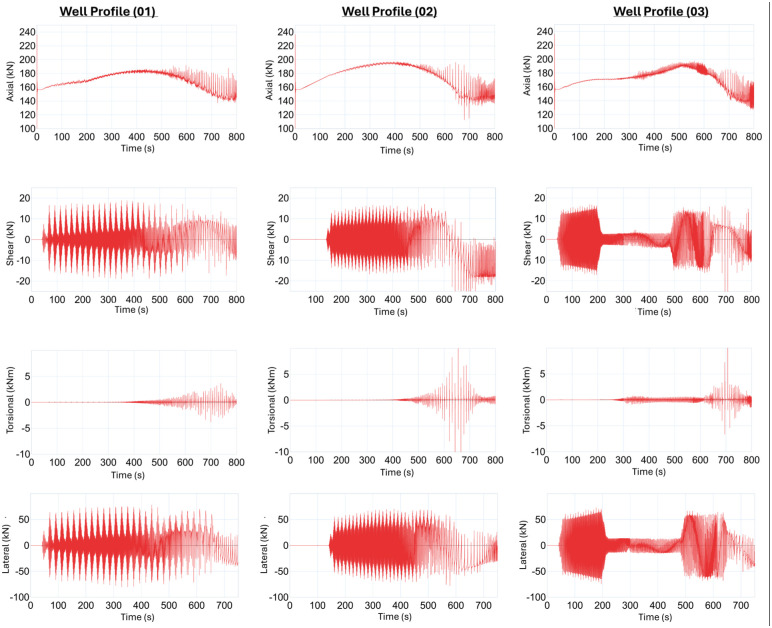
Internal force and torque variation along the build section for different well profiles.

Figure 15 shows the internal forces and torque variation along the build section of the drill string segment that is 40 m before the bit. Since these results show internal loads from the spring elements between rigid segments, the variation between positive and negative values indicates the exchange of energy within the drill string due to cyclic windup and release during drilling vibrations. These results show that a gradual build section in well profile #1 has the lowest vibration levels. Moreover, when looking at the shear and bending stress history results of profile #3, a clear high-stress region can be observed when the string segment passes through the first and second bend sections.

One of the main advantages of simulating the dynamics of the drill string tripping through the build section is its ability to generate the internal stress history at each point of the well profile. Experimentally measuring these results is impractical in an industrial application due to the harsh downhole conditions and limited communication bandwidth/methods. Hence, simulation models are the most feasible method to accurately predict the internal stress of downhole components, which can be used for further analysis, such as fatigue prognosis.

**Figure 16.**
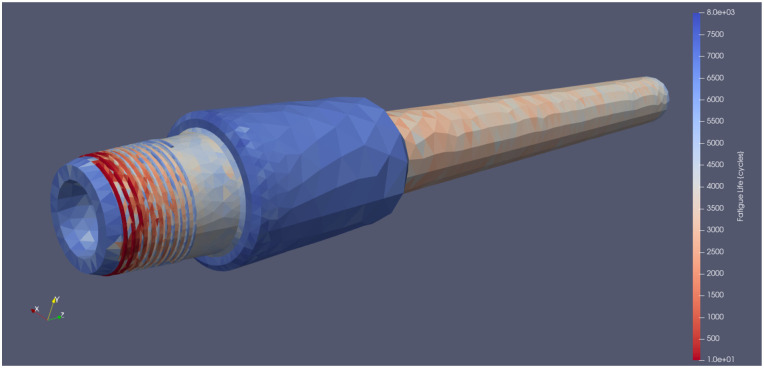
Sample fatigue analysis results.

Using the data from the previous analysis, a MNV fatigue simulation was carried out using the SalomeMeca^®^ open-source FE analysis software package. A sample of these analysis results is given in Figure 16. A 3D model of the drill pipe with threaded connections in an upset area was used in this fatigue prognosis. After performing a mesh sizing analysis, a Tetrahedra mesh with a minimum element size of 1 mm was selected. Force data obtained from the model shown in Figure 15 were applied to a point node, which was then connected to the threaded area using RBE3 elements.

From the fatigue analysis, the well profile #2 section can run the 800-s simulation window 12.8 times before failure. According to the FEA analysis, the failure of the components is most likely to be initiated near the threaded connections, which have the highest stress concentration due to their geometry.

The well profile #1 has a predicted lower fatigue life and would fail after 10.3 operational cycles of the 800-s simulation window. Even though profile #2 has a higher dog leg severity, the kick-off point in that profile is at a later stage and has a longer vertical section, which comparatively reduces the time the drill string is under high-stress loading. Due to this reason, profile #2 has a better fatigue life compared with profile #1. As expected, profile #3 had the lowest fatigue life and was predicted to fail after 5.9 completion of the given well profile.

In summary, this case study shows the potential application of the model in the planning stages of drilling operations. Moreover, as mentioned in the literature review section, drill pipes are replaced with significant useful operational hours remaining because of the inability to accurately predict fatigue. Hence, a model that can extract stress histories incorporating the complex contact conditions of the build section is motivated. Validation and field trials of the model will, in the future, increase the confidence of drilling engineers to better utilize the useful life of downhole drilling equipment without fearing unplanned tripping operations.

### 4.3. Case study—vibrational characteristics and fatigue analysis of the horizontal section

Downhole equipment, especially the measuring while drilling (MWD) tools, fail due to high vibrations.^12^ Hence, drilling companies are keen on understanding downhole dynamics and selecting the most suitable position for MWD tools and other sensitive equipment. This case study shows how the developed model can perform fatigue analysis for drill collar segments in the horizontal section of a deviated well.

**Figure 17.**
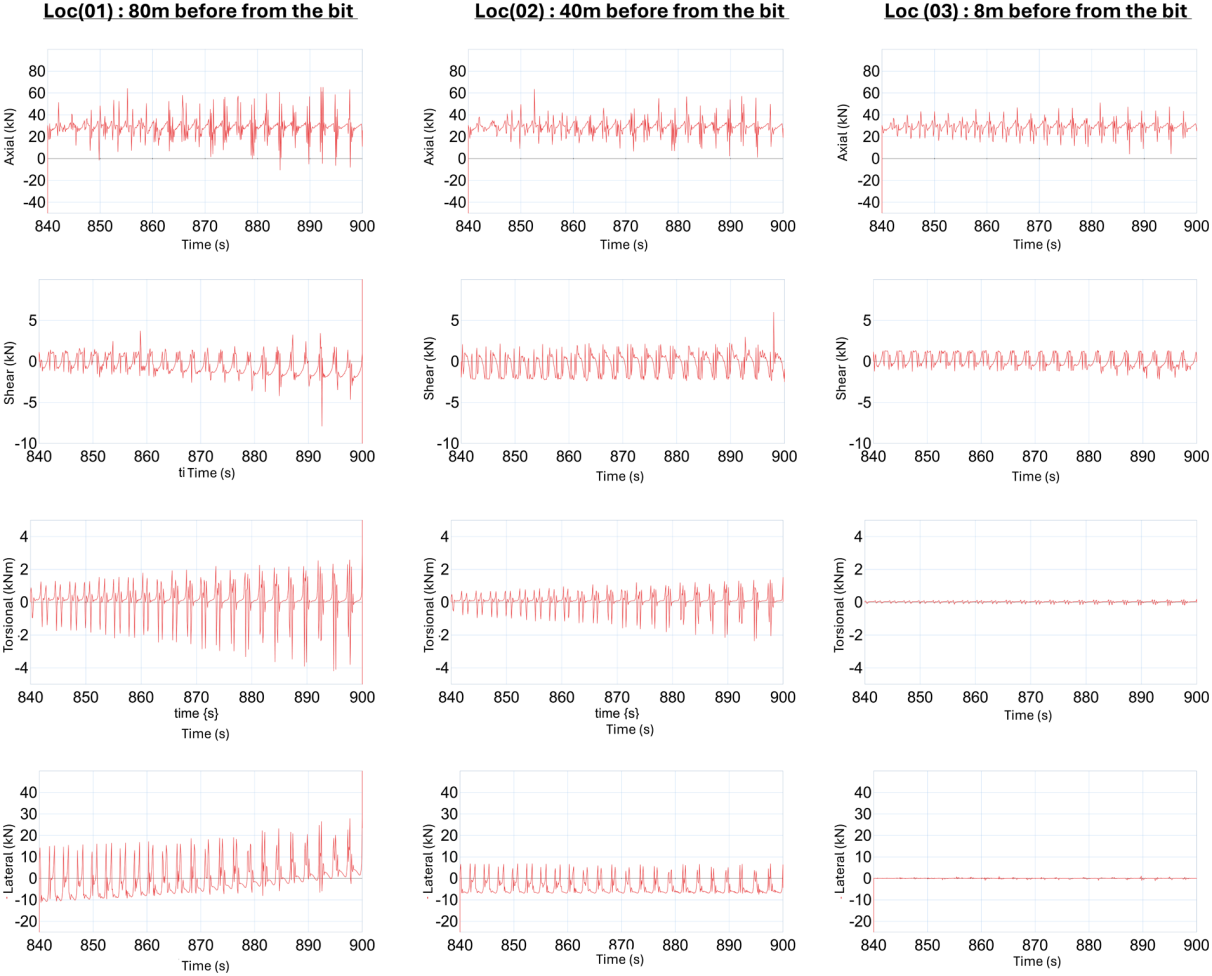
Internal force and torque variation for three different locations in the horizontal section.

The well profile #1 shown in Figure 14 was used for the dynamic analysis. The drill string was tripped into the well for the first 800 s, and the rotation speed was slowly increased to 50 r/mim, reaching steady-state operation conditions within the next 40 s. Afterward, the drill string internal stresses were then taken for a 60-s interval for three locations within the horizontal drill collar section. These locations are approximately 8, 40 and 80 m from the bit, and internal load information is graphed in Figure 17. It took around 2 h to run this 60-s interval using the BDF integrator with tolerances set to 1e-5.

In this simulation, the drill string was not subjected to bit bounce or whirling vibrations, and a sinusoidal function was applied to emulate bit vibrations. The results show that higher vibration levels are observed at location 1 compared with the other two measured points. This is because location 1 is near the end of the build section and is more susceptible to the dynamics from frictional contact forces, which impede free movement and amplify vibrations. Moreover, when comparing the results between locations 2 and 3, the stress in the location closest to the bit shows the lowest, and this could also be due to the influence mentioned earlier.

The fatigue analysis was carried out similarly to the previous case study using a drill collar segment with a threaded section, and results predict 191, 625 and 677 h of operation before failure for locations 1, 2 and 3, respectively. Hence, based on the findings for this particular drilling operation condition, placing the MWD tool closer to the bit would increase its durability. However, as mentioned earlier, the deviated section has a significant influence, and these findings might differ as the length of the horizontal section increases with the drilling progression.

## 5. Conclusion and future work

Previous drill string dynamic models have not included the effects of complex intermittent contact behavior within the build section, limiting the accuracy of predictions in horizontal sections. A new model was developed using an extensible vertical drill pipe, an improved friction model, accurate top drive and bit boundary conditions, and enhanced contact algorithm with a stiction/friction model. This model calculates internal stress and vibration characteristics during drilling, including in the build section and during tripping operations. The special beginning and end segment models developed in this study enable the lengthening of the drill string and guiding the bit as drilling progresses or during tripping operations.

The first case study demonstrated the ability of the developed models and the enhanced friction model to efficiently predict whirling vibrations. In the second case study, the progression of drilling of different well profiles was analyzed using a MNV fatigue simulation carried out from internal stress history data obtained from drill string MBD simulations. This study demonstrated how the derived model can be utilized during well planning stage to identify the most suiable well profile and showed that dog leg severity and the kick-off point influence the stresses and fatigue life of downhole components. The third case study analyzed fatigue damage to downhole equipment in the horizontal well section and showed that dynamics from the build section can affect the fatigue life of components in the horizontal section.

In the future, the authors plan to develop a simplified model to implement a digital twin methodology similar to Don and Rideout^79^ for deviated wells, enabling fatigue predictions based on operational conditions. Such a model will identify operational conditions such as harsh vibration and normal drilling and collect internal stress history in a representative window. This stress information will later be processed to calculate the fatigue life of downhole components and provide data for drilling engineers to make informed decisions.

The authors recognize that validation against actual drilling measurements is important for industrial adaptation and are currently seeking a high-fidelity downhole vibration data set. The case studies demonstrate that the proposed model improves current state-of-the-art simulation capabilities with parameters that map to physically meaningful assumptions. This model, after validation through field experiments, would enable improvements in well design, increased operational life of downhole equipment and reduction in unplanned tripping operations, thereby improving drilling efficiency and reducing unanticipated costs.
